# Phenotypic plasticity and modularity allow for the production of novel mosaic phenotypes in ants

**DOI:** 10.1186/s13227-015-0031-5

**Published:** 2015-12-01

**Authors:** Sylvain Londe, Thibaud Monnin, Raphaël Cornette, Vincent Debat, Brian L. Fisher, Mathieu Molet

**Affiliations:** UMR 7618 Institute of Ecology and Environmental Sciences of Paris, Sorbonne Universités, UPMC Univ Paris 06, 7 quai St Bernard, 75 252 Paris, France; Département Systématique et Évolution, Muséum National d’Histoire Naturelle; CNRS UMR 7205, Institut de Systématique, Evolution, Biodiversité, Paris, France; Department of Entomology, California Academy of Sciences, Golden Gate Park, 55 Music Concourse Drive, San Francisco, CA 94118 USA

**Keywords:** Queen, Intercaste, Developmental recombination, Caste evolution

## Abstract

**Background:**

The origin of discrete novelties remains unclear. Some authors suggest that qualitative phenotypic changes may result from the reorganization of preexisting phenotypic traits during development (i.e., developmental recombination) following genetic or environmental changes. Because ants combine high modularity with extreme phenotypic plasticity (queen and worker castes), their diversified castes could have evolved by developmental recombination. We performed a quantitative morphometric study to investigate the developmental origins of novel phenotypes in the ant *Mystrium rogeri*, which occasionally produces anomalous ‘intercastes.’ Our analysis compared the variation of six morphological modules with body size using a large sample of intercastes.

**Results:**

We confirmed that intercastes are conspicuous mosaics that recombine queen and worker modules. In addition, we found that many other individuals traditionally classified as workers or queens also exhibit some level of mosaicism. The six modules had distinct profiles of variation suggesting that each module responds differentially to factors that control body size and polyphenism. Mosaicism appears to result from each module responding differently yet in an ordered and predictable manner to intermediate levels of inducing factors that control polyphenism. The order of module response determines which mosaic combinations are produced.

**Conclusions:**

Because the frequency of mosaics and their canalization around a particular phenotype may evolve by selection on standing genetic variation that affects the plastic response (i.e., genetic accommodation), developmental recombination is likely to play an important role in the evolution of novel castes in ants. Indeed, we found that most mosaics have queen-like head and gaster but a worker-like thorax congruent with the morphology of ergatoid queens and soldiers, respectively. Ergatoid queens of *M. oberthueri*, a sister species of *M. rogeri*, could have evolved from intercastes produced ancestrally through such a process.

## Background

Darwinian theory and its subsequent developments have led to a good understanding of the gradual adaptation of quantitative traits. Nonetheless, the origin of discrete novelty remains poorly understood, despite evidence that many qualitative shifts have occurred during evolution [1]. Numerous examples suggest that novel phenotypes can result from the reorganization of existing ones, as exemplified in particular by research on homeotic genes [2, 3]. Important novelties such as animal eyes and tetrapod limbs also evolved through modifications of complex regulatory circuits already present in ancestors (“deep homology,” [4]). In the ant genus *Pheidole*, Rajakumar et al. [5] showed that the convergent evolution of super soldiers is due to the induction of an ancestral developmental potential. Such examples of evolutionary tinkering show that natural selection acts by modifying structures that are already available (i.e., *combinatorial evolution*, [6–10]; Table 1). Here we explore the mechanism of morphological novelty production in ants based on the recombination of alternative phenotypes (winged queens and wingless workers) and involving phenotypic plasticity and modularity.

**Table 1:** Glossary

Notions	Definition	Main references
Combinatorial evolution	Evolution by reorganization of preexisting elements. Different subunits are rearranged, and can be deleted, duplicated, and moved in various ways. This occurs not only at molecular level [112] but also at higher phenotypic levels. Typical examples of such process are heterochrony (changes in the timing of a developmental process) and heterotopy (changes in spatial location of a developmental process) [113]. Such evolutionary changes are likely to result from selection of mutations in cis-regulatory elements within particular gene regulatory networks [45]	Jacob [6]; McGinnis and Krumlauf [112]; Maeshiro and Kimura [114]; West-Eberhard [9]; West-Eberhard [10]
Developmental recombination	Reorganization of ancestral phenotypic traits in a particular individual, before genetic accommodation has fixed the phenotype in the population. The process has also been termed ‘chimeric’ or ‘somatic’ recombination	West-Eberhard [9]; West-Eberhard [10]; see also Davidson [14]; Ray [15]; Raff and Kaufman [16]
Genetic accommodation	Selection on standing genetic variation that molds the plastic response of a phenotypic trait. This occurs when the developmental-genetic system is sensitized, because genetic variation becomes exposed as phenotypic variation when the organism encounters a different environment	Waddington [18]; Suzuki and Nijhout [19]; Moczek [20]; Nijhout and Suzuki [21]; Suzuki and Nijhout [22]
Mosaic phenotype	Phenotype recombining within single individual traits that are normally found in distinct individuals	Wheeler and Weber [71]; Yang and Abouheif [109]

Some authors suggest that evolutionary novelty may result from the recombination of ancestral phenotypes following changes in the timing or the location of preexisting developmental processes [1, 5, 9–13]. This mechanism is referred to as *developmental recombination* ([9, 10, 14–16]; Table 1). For example, the recurrent evolution of limnetic vs. benthic forms in stickleback (*Gasterosteus aculeatus*) could have resulted from the altered expression of adaptive traits evolved in ancestral populations, emerging when these traits were expressed alternatively at different times during the life cycle [9, 17]. It has been proposed that evolution by developmental recombination may occur following three steps [10]: (1) Initially, a population consists of individuals that respond differently to some environmental and genomic inputs, because of genetic variation that either encodes distinct fixed traits, epistatic relationships, or distinct sensitivity levels to environmental factors (plastic response). (2) Some individuals are affected by a new genetic or environmental input that causes a reorganization of their phenotype because some phenotypic subunits differ in their responsiveness to the new input (developmental recombination). (3) If the resulting change in phenotype has a positive effect on fitness, it will be favored through selection of genetic factors involved in the plastic response. Selection therefore can lead to further changes in the regulation of development (frequency, timing, and circumstances of the new response) and the characteristics of new traits expressed as differences in morphology, behavior, physiology, etc. [10] (See notion of “*genetic accommodation*,” [9, 18–22]; Table 1). This model of evolution by developmental recombination is based on the association of two widespread characteristics of living organisms: plasticity and modularity.

Phenotypic plasticity is the ability of a single genotype to produce alternative phenotypes in response to different environmental factors [9, 23, 24]. It results from the fact that the environment not only filters phenotypic variation but may also induce it [9, 13]. Some environmental cues can regulate gene expression and determine which structures will develop. When there is selection for the genetic ability to respond to environmental cues, phenotypic plasticity becomes adaptive [25]. Phenotypic plasticity can range from a subtle adjustment in growth rate to complete polyphenism involving the production of discrete alternative phenotypes [26]. The plastic response to environmental factors can be continuous (reaction norms) or discrete (polyphenisms) [27, 28]. Classical examples of phenotypic plasticity include the adjustment of biomass allocation to leaf tissue in plants in response to light intensity [29], the development of a more robust head capsule, and a stronger bite in grasshoppers that have experienced hard food during ontogeny [30, 31], and the development of morphological castes in social hymenoptera [32]. Phenotypic plasticity may play an important role in evolution because responsiveness to the environment affects the strength of selection under alternative environmental circumstances and therefore the intensity of drift and the accumulation of standing genetic variation [33]. Such standing variation is available to be acted upon by genetic accommodation and the canalization of an initially environmentally induced trait can evolve rapidly in response to selection [34]. Experimental approaches confirm that some adaptations derive from characters that were environmentally induced in ancestral populations [10, 11, 35, 36]. Because phenotypic plasticity involves alternative phenotypes, it may also facilitate combinatorial evolution by increasing the number of distinct versions of each module, and thus the number of building blocks available for developmental recombination (e.g., [9, 37, 38]).

Modularity is a universal property of organisms resulting from the branching nature of development [39]. Modules are units, the subparts of which are strongly integrated by numerous genetic, developmental, or functional interactions [40], but are more weakly connected to other modules [41]. Modularity at the anatomical scale is underpinned by the modularity of developmental processes including interactions among proteins and gene regulation [41–44]. Indeed, regulatory gene interactions form a network with multilevel modular subcircuits. Although most proteins regulating development have pleiotropic effects, the timing of their expression is specifically regulated in different spatial domains by cis-regulatory elements within particular gene regulatory networks (GRNs) [45]. Genetic input affecting particular cis-regulatory modules can thus affect some phenotypic subparts independently of others. As a result of this modular architecture, some mutations can cause gains, losses, or redeployments in other contexts of a function or a morphological structure controlled downstream of the mutations, without affecting other components of the phenotype [46]. A deep level of anatomical modularity is governed by Hox genes that control body plan morphogenesis along the anterior–posterior axis. For instance, the control of forelimbs and hind-limbs by different phases of Hox gene expression regulated by separate cis-regulatory enhancer elements has allowed birds and bats to evolve wings as well as legs [47]. At the phenotypic level, because the subparts that form one module experience the same selective pressure, they co-evolve semi-independently from subparts of other modules [9, 41]. A consequence of this independence is that modules such as arthropod imaginal disks can develop numerous times along the body plan or be deleted independently of other traits [2, 48, 49]. For instance, the Ultrabithorax gene prevents wing development from the imaginal disk of the third thoracic segment by repressing cis-regulatory modules at multiple nodes of the wing patterning GRN [50]. This property allows for the reorganization of different phenotypic building blocks, i.e., evolution by developmental recombination [9].

Ants combine an extreme polyphenism (queen and worker castes) with a high modularity typical of arthropods. They are a highly diversified clade with various morphological castes [51–53 for an overview of this diversity]. The ancestral and most common caste system features winged queens that perform aerial dispersal and reproduction, and wingless workers that are involved in brood care, foraging, defense, cleaning, and construction of the nest. Accordingly, queens have a fully functional reproductive system with a spermatheca, a large articulated thorax with flight sclerites, large wing muscles and wings, and three ocelli. In contrast, workers have an atrophied reproductive system, a reduced fused thorax lacking wings and flight muscles, and generally no ocelli [51, 52]. In many species, workers may vary in size but do not differ in shape (minor vs. major workers). However, many species have evolved new castes, such as permanently wingless queens called ergatoid queens [54–59] and soldiers that differ from workers in the relative growth of some body parts [60].

Typically, queen and worker castes are determined mainly by the environment [61, 62]. Environmental factors (e.g., food quality and quantity, temperature and queen pheromones) affect hormonal secretions (mainly juvenile hormone) that induce physiological and cellular responses, ultimately resulting in a developmental switch toward either caste [61, 63]. Switches are determined by several factors such as hormone titers and timing of tissue sensitivity to hormones, and result in caste-specific patterns of gene expression possibly mediated by DNA methylation [64–66]. Regarding wing polyphenism, Abouheif and Wray [67] have shown that wingless worker and soldier phenotypes are produced through interruptions in the gene regulatory networks yielding wings in queens in the imaginal wing disks of larvae. Interruptions occur at different points in the network depending on species. Caste determination in some species also involves various degrees of genetic control [68].

Molet et al. [69] proposed that some new castes in ants may have evolved via developmental recombination of existing castes. Indeed, wingless queens and soldiers appear to be *mosaics* (Table 1) of winged queen and worker morphologies [60, 69]. This hypothesis implies that environmental or genetic inputs can generate new phenotypes, a known phenomenon in ants. In addition to the discrete queen-worker dichotomy, some authors have described individuals that are neither queens nor workers, called intercastes [54, 70–75]. Intercastes are rare, anomalous adults with various morphologies, visually ranging from almost similar to winged queens to almost similar to workers. Although generally not winged, intercastes often have simplified flight sclerites and on occasion wing stubs. They also may have a spermatheca and one to several ocelli. Thus, intercastes seem to be mosaic phenotypes recombining queen and worker traits. Importantly, they survive as adults, although their behaviors are unstudied. They can be produced when unusual genetic and environmental inputs exceed the buffering capacities of development. This could disturb signaling pathways upstream of some caste-specific GRNs, leading to anomalous gene expression during the ontogeny of the corresponding morphological structures. As a result, modules of the developing larvae do not consistently follow queen and worker pathways. More specifically, departure from normal developmental processes could be the consequence of changes (gains, losses, or modifications) in linkages within GRNs caused by the evolution of cis-regulatory elements (CREs). This includes the co-option of new transcription factor inputs by mutations in existing CREs [76, 77], the co-option of transposable elements as new CREs [78], the loss of transcriptional inputs in existing CREs [79], and the remodeling of CREs [80].

Intercastes have been described in about 20 species [74] and are likely to be taxonomically widespread. These mosaic individuals probably either go unnoticed due to their rarity or are discarded by researchers because of their abnormal features. Because some intercastes look morphologically similar to ergatoid queens, we suggest that they represent an early step in the evolution of ergatoid queens, before the selection of genetic factors involved in the induction of their phenotypes has fixed a particular phenotype (i.e., genetic accommodation). The emergence of a new caste from environmentally induced anomalies followed by genetic accommodation has also been proposed for the evolution of super soldiers in *Pheidole* [5]. Accordingly, studying intercastes, and more generally, developmental mechanisms allowing for the production of mosaic phenotypes, will contribute to our understanding of caste evolution.

The intuitive concept of mosaicism has allowed for the description of intercastes based on striking, discrete traits such as the presence or absence of wings and ocelli and of a broad or narrow thorax [73, 74, 81, 82]. However, no quantitative measure of mosaicism has been performed, and consequently phenotypes with less obvious mosaicism have likely been ignored. This means intercastes as described in the literature probably only represent a fraction of the existing range of mosaicism. Indeed, a continuous range of mosaic phenotypes, ranging from worker-like to queen-like, probably exists. Intercastes following the classical definition may only be highly striking cases of mosaic phenotypes (i.e., clearly intermediate between workers and queens), and less distinctive individuals at the extreme of this continuum (i.e., more worker-like or more queen-like) may remain undetected by researchers. Therefore, we propose a new procedure based on morphometric data to quantify the degree of mosaicism and precisely describe the range of combinations among queen and worker modules. We test whether individuals initially identified as intercastes based on discrete characters are effectively mosaics for quantitative morphometric traits, and whether additional mosaic individuals have previously been overlooked.

**Fig. 1:**
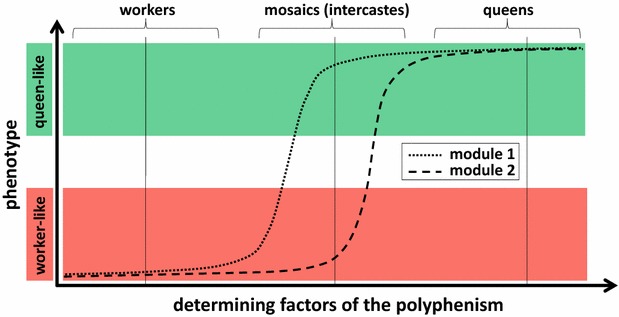
Hypothesis for the developmental origin of mosaic phenotypes. Each larva may develop into a queen or a worker depending on determining factors experienced during its ontogeny. In ants, determining factors are mainly environmental, although genetic influences have also been found in some species. If different modules respond differently to determining factors, intermediate levels of these factors induce the production of mosaic individuals with queen-like morphology for some modules and worker-like morphology for others. For instance, the intercaste depicted here (vertical line for intermediate value of determining factors) has a queen-like module 1 but a worker-like module 2

In this study, we do not investigate the genetic determinants of the evolutionary changes leading to intercastes. Many mutational mechanisms may cause these changes. Instead, we analyze the final product of developmental processes, i.e., phenotypes. That is, we focus on the level directly visible to natural selection. We propose that mosaic phenotypes may be produced in ants because the latter exhibits a high degree of modularity and phenotypic plasticity. Indeed, if different modules have different response thresholds to the same inducing factor, mosaic phenotypes may be generated by intermediate levels of factors inducing differential responses among modules (Fig. 1). In most cases, normal workers and winged queens would be produced because the levels of inducing factors are far above or far below the response thresholds of all modules. However, on the rare occasions where intermediate levels of inducing factors are experienced, some modules within one larva may develop as in workers, whereas others may develop as in queens, thereby resulting in a mosaic individual combining worker and queen phenotypic traits. This hypothesis has two corollaries that we test in this study: (1) Modules have distinct patterns of variation in response to caste-determining factors. This causes a differential response among modules for intermediate values of caste-determining factors. (2) The range of possible mosaic phenotypes is strongly constrained by the distinct patterns of variation of the different modules. Finally, we discuss the developmental origins of mosaic phenotypes and their significance regarding the model of developmental recombination and the multiple evolutions of ergatoid queens and soldiers in ants.

## Methods

### Biological model

We used the ant *Mystrium rogeri* Forel, 1899 (Amblyoponinae) as it is an ideal model to study the production of intercastes and the evolution of new castes. This species erratically produces intercastes [83, 84], and the genus *Mystrium* includes both species with winged queens (*M. rogeri* and *M. camillae*) and species with ergatoid queens (*M. oberthueri* and *M. voeltzkowi*) [84]. Accordingly, evolutionary transitions from winged queens to ergatoid queens have occurred in this genus in the past, and may be ongoing in extant *M. rogeri* populations.

### Colony collection and choice of samples

We collected 60 colonies of *M. rogeri* in a rainforest in Andrambovato, Madagascar, in November 2011 and January 2013. Specimens from each colony were deposited at the ant collection of the California Academy of Sciences, and can be accessed through AntWeb [53; list of colonies in Table 2]. Each colony contained an average of 6.1 (SD ± 4.2) queens (one queen and several unmated gynes), 40.4 (±20.5) workers, and 15 (±10.4) males. Intercastes were identified under a stereomicroscope based on discrete characters mentioned in classical literature about intercastes (e.g., [74, 75]). Winged queens of *M. rogeri* have fully developed flight sclerites and three ocelli that are lacking in workers [84]. Consequently, individuals were classified as intercastes if (1) they had one ocellus, two ocelli, or three reduced ocelli or (2) they were wingless but had wing stubs or relatively unfused flight sclerites. Out of the 60 colonies, 19 (31 %) had 2.4 (±1.2) intercastes. In total, among the 2798 female individuals examined in the 60 colonies, 1.4 % were intercastes. However, one must keep in mind that the definition of intercastes only allows identifying the individuals that are strikingly intermediate between queens and workers, but not all intermediates. Accordingly, we did not rely on this a priori classification of intercastes in our morphometric study, but used unbiased methods instead (see “Queen-likeness index” section below). Since a morphometric analysis of all colonies was technically not possible, we chose to study three complete colonies that had several intercastes (colonies BLF27637, BLF27647, and BLF27654, with a total of 19 intercastes, 16 queens, and 100 workers). In addition, we increased our sample of phenotypes intermediate between queens and workers by including 18 additional intercastes found in 10 out of the remaining 57 colonies. To control for colony effect, we added one nestmate worker and one nestmate queen (when available) per additional intercaste. Our final sample thus consisted of 29 queens, 37 intercastes, and 124 workers (Table 2).

**Table 2:** Number of queens, intercastes, and workers from each colony in our sample

Colony	Queens	Intercastes	Workers
BLF27637^a^	1	9	21
BLF27647^a^	2	5	32
BLF27654^a^	13	5	47
BLF27635	5	7	9
BLF27649	4	1	4
BLF27652	1	2	2
BLF30540	0	1	1
BLF30543	1	0^b^	1
BLF30551	0	1	1
BLF30553	1	1	1
BLF30557	1	1	1
BLF30558	0	1	1
BLF30574	0	3	3
Total	29	37	124

^a^Colonies studied in their entirety

^b^One intercaste was initially present in colony BLF30543 but was damaged during handling

### Morphometrics

**Fig. 2:**
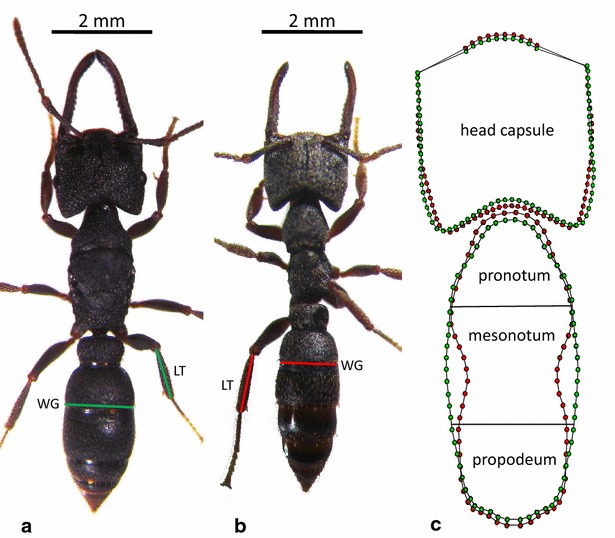
Morphology of a queen (**a**) and a worker (**b**) of *Mystrium rogeri*. Queens are larger than workers. The thorax of a queen is enlarged and made of distinct subunits, whereas the thorax of a worker is fused. Queens have three ocelli, whereas workers have none. Queens have a relatively larger gaster. LT and WG: length of tibia and width of gaster. **c** Digitalized outlines and semi-landmarks after sliding for queens (*green circles*) and worker (*red circles*). The differences in head morphology between both castes have been amplified by 10 %

We worked on six developmental modules corresponding to anatomical structures developing from distinct tagma, metameres, or imaginal disks during ontogeny. We took 2D measurements on four modules (head capsule, pronotum, mesonotum, and propodeum) and linear measurements on two modules (length of tibia and width of gaster). These modules are relevant for our investigation because they are rigid, unarticulated, and dimorphic between winged queens and workers (Fig. 2). Combining 2D and linear modules in the same analyses is not a problem because we did not compare shapes and sizes directly. Instead, we computed a dimensionless index of polyphenism as our main variable (see “queen-likeness index” here after).

### 2D shape analysis

We used geometric morphometrics to extract shape information from the four modules analyzed in 2D. Head, thorax, and legs were separated and laid flat. Photographs were taken under a stereomicroscope and each module was photographed twice to take into account small deviations caused by optical biases such as lighting variations or position relative to camera lens [85]. We then digitalized the photographs using TPSDig2 software [86]. Measurement error was computed using Procrustes ANOVA [87] based on the two photograph sessions. Polyphenism induces large shape differences between castes, so identifying numerous homologous anatomical landmarks for shape analysis is not possible. Therefore, we studied outlines using the sliding semi-landmarks method [88]. For head shape, we defined one set of semi-landmarks along the edges of the head capsule and another set along the edges of the clypeus in dorsal view. For thorax shape, we digitalized one set of semi-landmarks along the edges in dorsal view. The thoracic configuration was then split into three partial configurations: pronotum, mesonotum, and propodeum (Fig. 2c). Semi-landmarks were aligned using the minimum Procrustes distance criterion [89, 90]. For each individual and module, we calculated an average configuration based on the two photographs. We focused our analysis on symmetrical variance. In this way, we reflected lateral semi-landmark configurations across the symmetry axes using the “object symmetry” procedure [91]. The shape component of each configuration was extracted using the Procrustes superimposition method [85, 87, 92, 93]. All modules were first rescaled with their own unitary centroid size in order to extract shape information. Rescaled configurations were then superimposed and rotated around their centroid so as to minimize the sum of squared distances between corresponding semi-landmarks. All analyses were performed in R 3.0.1 using routines from the geomorph library [94] and function from Claude [85].

### Queen-likeness index

**Fig. 3:**
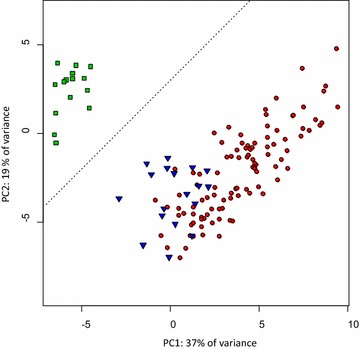
Distribution of morphology without caste a priori. Distribution of all individuals from the three complete colonies (BLF27637, BLF27654, and BLF27647) in the space defined by the first two components of the principal component analysis on pooled data from the six modules (shape and centroid size of head, pronotum, mesonotum, and propodeum; gaster width; tibia length). As expected in ants, two groups were detected (one on each side of the *dotted line*) and used as references to determine the queen/worker axis for each module and calculate their queen-likeness. Affiliations to queen (*green squares*), worker (*red circles*), and intercaste (*blue triangles*) phenotypes as classically determined were added a posteriori to the figure and were not used for the statistical analysis

Mosaicism assessment requires an index that quantifies how similar each module is to typical queen or worker morphologies. Accordingly, for each module of each individual, we computed a queen-likeness index. In order to define typical queen and worker morphologies as objectively as possible, individuals were assigned to the queen or worker caste without any a priori visual categorization. Instead, we relied on the typical bimodal distribution of the phenotypes found in colonies. For this purpose, we performed a Principal Component Analysis (PCA) on the pooled data from the six modules (shape and centroid size for the four modules measured in 2D, and size for the two modules measured in 1D). The PCA only included individuals from the three complete colonies (BLF27637, BLF27647, and BLF27654) and not our entire sample, as we wanted to avoid a bias in the distribution of individuals caused by the artificial increase of intercaste frequency in our entire sample. As expected, PCA showed that the colonies consisted of two groups of individuals (Fig. 3), that we defined as queens and workers.

The second step was to generate a queen-worker axis for each module. This axis was computed using queen and worker affiliations as defined by the PCA. The data from the two modules measured in 1D could be used directly to generate two queen-worker axes. The data from the four modules measured in 2D required a reduction of dimensionality: we used Between Group Analyses (BGA) [95, 96] that maximize between-groups variance, and projected the results on the first component to generate four additional queen-worker axes.

The final step was to project our full dataset (the three complete colonies plus the additional samples) onto these axes. Then, in order to standardize the range of variation of each module from 0 for the most worker-like morphology to 1 for the most queen-like morphology, we shifted and rescaled the projected data. Each queen-likeness value V was, therefore, transformed as V′ = (V − Vmin)/(Vmax − Vmin).

Two alternative methods to compute the queen-worker axes were rejected. The first axis of a principal component analysis is not suitable because it does not maximize variance between groups. The first component of a linear or quadratic discriminant analysis not only maximizes the discrimination between groups, but also dramatically distorts intra-group variances by transforming relations among initial variables [97]. This effect could have biased the among-groups measure of mosaicism because our mosaicism estimator is the variance among queen-likeness indexes of each module within individuals (see “Mosaicism” section below).

### Profiles of variation

The hypothesis that mosaicism originates from differential responses to the caste-determining factors among modules implies that each module shows a specific pattern of variation along the worker-queen axis that we call a ‘profile of variation.’ Profiles of variation were obtained by plotting the queen-likeness of each module against a reference variable. We chose to use individual body length (sum of head and thorax length extracted from 2D configurations) as a reference variable because it is both methodologically independent from queen-likeness and illustrates queen-worker polyphenism well because queens are larger than workers [84]. Gaster length was not included in this reference variable because the gaster is an articulated structure that can be contracted or extended considerably depending on nutrition, reproduction, and preservation. Profiles of variation may be considered allometric relationships between an index of shape and body size.

In order to test whether profiles of variation differed among modules, we fitted a parametric model with queen-likeness data for each module using the R package ‘grofit.’ Given this model, we then extracted the individual body length for which queen-likeness of this module equals 0.5. We called this individual body length the ‘transition point.’ Differences in transition points among modules were tested using pairwise comparisons of mean transition points computed from 1000 bootstrap samplings [98].

### Mosaicism

Mosaicism is the degree to which a phenotype recombines modules that normally occur in alternative phenotypes. For instance, an individual with a queen-like head and a strongly worker-like pronotum has a higher level of mosaicism than another individual with a queen-like head and a slightly worker-like pronotum. We computed mosaicism as the standard error of queen-likeness indexes for the six modules, i.e., the dispersion of queen-likenesses among modules. We compared the average mosaicism in queens, workers, and intercastes using Kruskal–Wallis tests. In order to visualize the link between mosaicism and the different profiles of variation among modules, mosaicism was also plotted against individual body length. Slope significance for queens, intercastes, and workers was tested against zero using a bootstrap procedure (1000 samples). In order to verify that mosaicism patterns were not artifacts caused by the queen-likeness computation method, we re-computed mosaicism following inter-individual permutation of queen-likeness for each module. We then tested whether mosaicism patterns disappeared; if so, we concluded there was no methodological artifact.

### Allometry

A preliminary analysis showed that shape of head, pronotum, mesonotum, and propodeum presented significant allometric relationships with centroid sizes (bootstrap procedure as advised by [99]: P < 0.01), except in queens where a significant allometric effect was only found for pronotum (P = 0.049) (we used standardized major axis regression in order to take into account errors in both queen-likeness indexes and size measurements, [100]). These allometric relationships mean that a proportion of the variance observed in our shape data (and consequently in queen-likeness) is explained by the size of the modules. We did not correct queen-likeness for these allometric effects because this would have affected the profiles of variation, which are themselves allometric relationships between the queen-likeness of each module and individual body size.

## Results

### Queen-likeness

Procrustes ANOVA showed that measurement errors were almost 10 times smaller than inter-individual variation (11.4 % for head shape, 0.4 % for pronotum shape, 2.1 % for mesonotum shape, 4.5 % for propodeum shape, 10.1 % for leg length, and 2.2 % for gaster width), confirming that our measurement methods were appropriate and that estimates of shape variances were not compromised by measurement error. Although significant, colony effect ranged between 2.2 and 7.5 times smaller than the effect of caste on the queen-likeness of different modules.

**Fig. 4:**
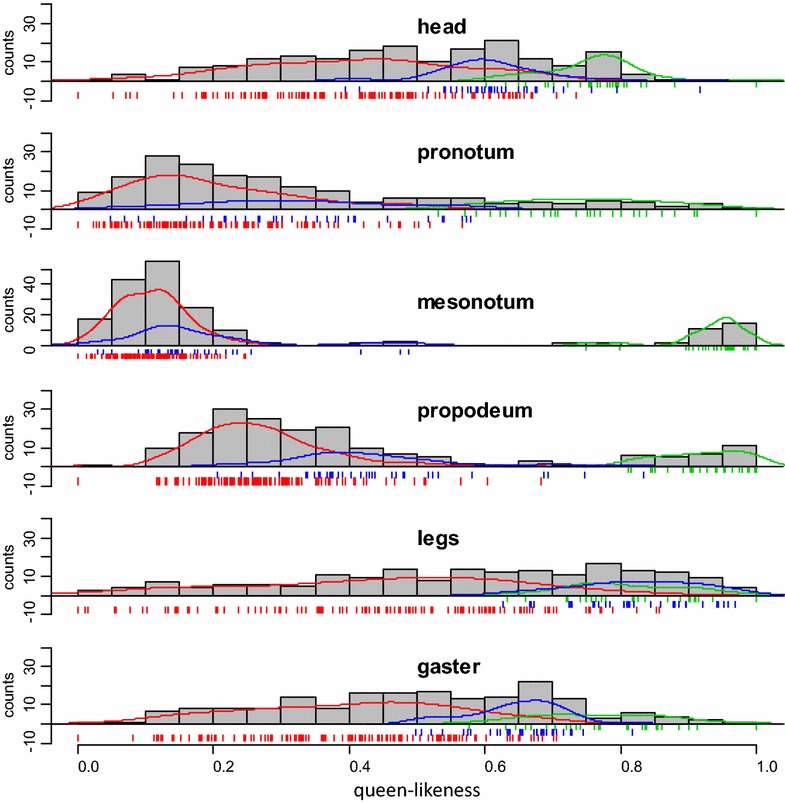
Distribution of queen-likeness for each module between groups. *Green* queens; *blue* intercastes; *red* workers. *Gray histograms* represent total distribution without caste distinction. Queen-likeness of intercastes was different from and intermediate between queens and workers except for legs, which did not differ from queens

We compared queen-likeness distributions between groups according to a priori assignation. Queen-likeness was significantly different between queens and workers for each module, with an overlap for head, legs, and gaster (Fig. 4). Queen-likeness of intercastes was different from and intermediate between queens and workers (Kruskal–Wallis rank sum test: P < 10^−6^), with the exception of legs, which did not differ from those of queens (Chi^2^ = 0.44, P = 0.51). The three thoracic modules of intercastes were more similar to workers than to queens and overlapped with workers (Fig. 4). Workers were significantly more variable than queens for all modules (Bartlett test: P < 10^−3^) with the exception of pronotum (K^2^ = 0.12, P = 0.73) and mesonotum (K^2^ = 0.24, P = 0.62). Workers were also significantly more variable than intercastes (Bartlett test: P < 0.05) except for pronotum (K^2^ = 1.75, P = 0.19). Intercastes were more variable than queens for mesonotum and propodeum (Bartlett test: P < 10^−6^) but not other modules.

### Profiles of variation

**Fig. 5:**
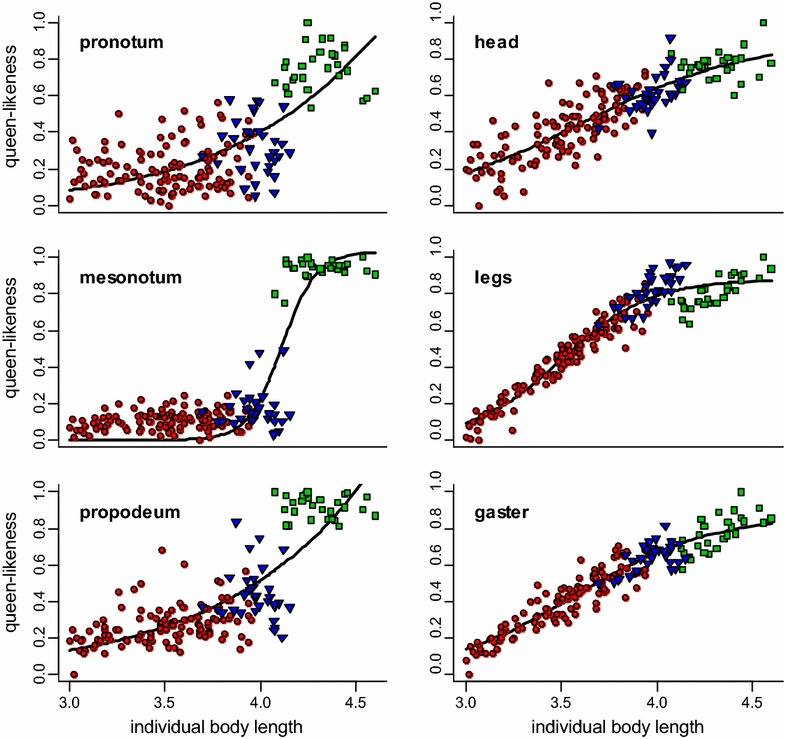
Profiles of variation for each module. *Green squares* queens; *blue triangles* intercastes; *red circles* workers. A sigmoid function was fitted to the data of each module. The three thoracic modules had a clear sigmoid relationship with body length, whereas head, legs, and gaster presented a more linear profile of variation when body length increased

The six modules had distinct profiles of variation (Fig. 5, 6a). Two types of profiles of variation could be distinguished graphically. As individual body length increases, queen-likeness of pronotum, mesonotum, and propodeum forms a long plateau at low values, and then increases late and suddenly to a plateau at high values. In contrast, queen-likeness of head, legs, and gaster increases earlier, at low values, and climbs continuously to plateau at high values. The two modules with the most divergent pattern were mesonotum and legs. The relative shape of their profiles of variation suggests that intercastes with an intermediate body length should have a worker-like mesonotum and queen-like legs. The transition points of the fitted parametric functions occurred at different body lengths among modules (pairwise comparisons on bootstrap distributions: P < 10^−6^) except for pronotum vs. mesonotum (P = 0.93) and head vs. gaster (P = 0.33). When body length increased, modules switched from worker-like to queen-like in the following sequence: legs, then gaster and head, then propodeum, and finally mesonotum and pronotum (Fig. 7).

**Fig. 6:**
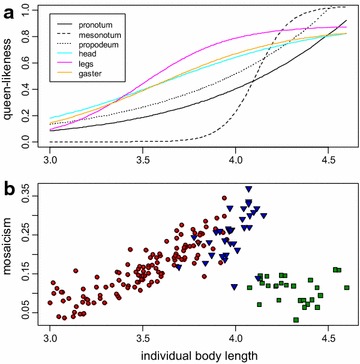
Consequence of the distinct profiles of variation among modules (**a**) on mosaicism (**b**). **a** Profiles of variation as a function of individual body length for the six modules. **b** Mosaicism as a function of individual body length for each individual (*green squares* queens; *blue triangles* intercastes; *red circles* workers). Mosaicism was low for both low and high body length, i.e., when all modules either showed a low or a high queen-likeness. As individual body length increased up to intermediate values, mosaicism increased from workers to intercastes (as expected from Fig. 1). There was no quantitative difference in mosaicism between the largest workers and intercastes

**Fig. 7:**
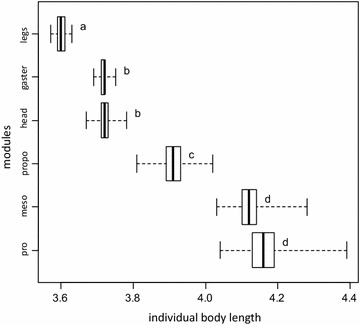
Distinct modules transition from queen-like to worker-like at distinct individual body lengths. This sequential transition restricts the range of mosaic phenotypes that can be produced. The transition point of a module is defined as the individual body length for which the profile of variation (depicted in Fig. 5) reaches a queen-likeness value of 0.5. *pro* pronotum, *meso* mesonotum, *propo* propodeum. *Different letters* indicate statistically different transition points (bootstrap procedure; 1000 resamplings)

### Mosaicism

Individual mosaicism was quantified as the standard error of queen-likeness for the six modules. Mosaicism was higher for intercastes than for queens and workers (respectively, Chi^2^ = 40.74, P < 10^−9^; Chi^2^ = 36.47, P < 10^−8^), and higher for workers than for queens (Chi^2^ = 16.98, P < 10^−4^) (Fig. 6b).

Plotting mosaicism against individual body length showed that mosaicism is continuous between workers and intercastes (Fig. 6b). Large workers were more mosaic than small workers (slope: 0.08; bootstrap P < 0.001), and as mosaic as intercastes. Mosaicism also increased with body length in intercastes (slope: 0.11; bootstrap P = 0.049) but not in queens (slope: −0.007; bootstrap P = 0.32). Inter-individual permutations of queen-likeness indexes erased this general pattern of mosaicism, proving that it reflected a biological reality and was not a methodological artifact.

The pattern of mosaicism against body length resulted from the variance among the profiles of variation of the different modules. Mosaicism was low for low body length, where all modules had a low queen-likeness. As body length increased, the parametric fits diverged among modules and mosaicism increased. Finally, at high body length, all modules had high queen-likeness and mosaicism became low again (Fig. 6).

## Discussion

Although they are rare (1.6 % of adult females), we obtained a reasonable number of intercastes by collecting 60 colonies of *M. rogeri*. Our geometric morphometric study of 29 queens, 37 intercastes, and 124 workers showed that the six modules (head, pronotum, mesonotum propodeum, legs, and gaster) grow differently as body length increases; i.e., they have distinct profiles of variation. This is in agreement with the hypothesis that the production of mosaics results from the differential responses of modules to the same inducing factors. Our results suggest that, for intermediate levels of inducing factors, some modules develop as in queens, while others develop as in workers, resulting in the production of mosaic phenotypes.

In our analyses, queen-likeness of each module was plotted against individual body length (Fig. 5). However, the significant correlation between queen-likeness and body length probably does not reflect a causal relationship, but rather a common response of both modules and body length to others factors that control polyphenic development. Body length is also a component of caste polyphenism, queens being larger than workers in many social insect species [52] including *M. rogeri* [84]. We used body length as a reference to compare the profiles of variation among several developmental modules, but the same divergence in scaling relationships among modules is expected with any other reference variable as long as this variable is also correlated with factors determining caste polyphenism. Indeed, the scaling relationships linking determining factors and body length necessarily affect the profiles of variation of the modules in the same way regardless of the reference used and thus do not qualitatively affect their divergence. The robustness of our results was confirmed using average queen-likeness as reference instead of body length (data not shown). Therefore, the divergence in scaling relationships among modules suggests that they respond differently to the factors driving caste polyphenism.

In ants, the factors driving development toward a worker or queen phenotype are primarily environmental [61, 63]. Yet, increasing evidence indicates that caste polyphenism can be controlled by genetic, genomic, and maternal factors to various extents [68, 101]; depending on the species, polyphenisms may range from fully environmentally induced to fully genetically determined. In the first case, profiles of variation as established in our procedure would be interpreted as reaction norms [27, 28] with an unknown rescaling function in the abscissa depending on the link between environmental factors and body length. In the second case, divergences in profiles of variation would represent pleiotropic effects among modules in response to various genetic inputs. Our experimental design cannot distinguish genetic components from environmental components. Both might be responsible for the observed effects. The results, therefore, suggest that mosaicism is generated by intermediate levels of determining factors, regardless of their genetic or environmental nature, inducing differential responses among modules.

Mosaicism increases gradually between workers and intercastes and abruptly falls in queens (Fig. 6b). This is in accordance with the progressive divergence among profiles of variation from low to intermediate body lengths, and their rapid convergence from intermediate to high body lengths (Fig. 6a). This suggests that the absence of mosaic phenotypes intermediate between intercastes and queens is not due to an incomplete sample, from which queen-like intercastes would be missing by chance. Rather, it results from the late and sharp increase of queen-likeness for the mesonotum (Fig. 6a), which dramatically reduces the window of sensitivity and leads to very queen-like intercastes. An obvious explanation for the late increase of queen-likeness for the mesonotum is that a queen-like mesonotum is an adaptation to flight that is energetically costly to produce [32]. Selection could allow small variations in thorax morphologies for workers and intermediate phenotypes, but canalize queen morphology by allowing the expression of an expensive, queen-like mesonotum only in individuals expressing queen-like modules, i.e., in perfect queen phenotypes. Without a markedly dimorphic mesonotum, mosaicism would probably decrease more continuously between intercastes and queens, following a reverse U-shape (i.e., increase then decrease) gradually connecting workers to queens. The gradual increase in mosaicism from low to intermediate body length shows that intercastes as described in the literature are only particularly noticeable mosaic individuals with striking qualitative characters among a continuous range of mosaics. Our quantitative approach leads to a better understanding of the developmental mechanisms underlying queen/worker polyphenism.

Using the profiles of variation of the different modules, we can predict which mosaic phenotypes can or cannot be produced in *M. rogeri*. Among modules, queen-likeness increased at different rates with body length; as a result, the transition points (body length at which queen-likeness exceeds 0.5) differed among modules (Fig. 7). As body size increased, legs were the first module to pass the transition point, followed successively by gaster and head, then propodeum, and finally mesonotum and pronotum. This sequential switch from worker-like to queen-like modules restrains the range of possible combinations. For instance, an individual with a queen-like pronotum is unlikely to have worker-like legs, gaster, or head. Okada et al. [82] found a similar sequential limitation in possible phenotypes in intercastes of the ant *Temnothorax nylanderi*. This sequence is likely shared among ants, but further investigation in other species is required.

Previous works suggested that plasticity and modularity allow for combinatorial evolution by reorganizing phenotypes through developmental recombination [9–11, 13, 69]. In social hymenoptera, it is known that the reorganization of phenotypes (i.e., production of intercastes) can be induced by artificially manipulating the environment or physiology of larvae [102–105]. However, for evolution by developmental recombination to occur, two steps are necessary. First, new combinations of characters must occur spontaneously in nature and be exposed to selection. The present study shows that this is the case in *M. rogeri*. Second, the production of new combinations must be heritable. Importantly, although polyphenisms and reaction norms are plastic responses to environmental factors (i.e., nonheritable factors), their features are genetically controlled, so they can evolve under selection [106, 107]. In addition, phenotypes that are environmentally determined can become genetically determined following adjustments in the sensitivity of development [108], an evolutionary process known as genetic accommodation [9, 19–22]. Since any mosaic phenotype is visible to natural selection through its direct fitness when fertile or its contribution to colony fitness when sterile, its production could increase as a result of selection on additive genetic factors that change the shape of the profiles of variation for some modules and expand the window of sensitivity allowing induction of this phenotype. At the molecular level, evolution by developmental recombination can rely on the selection of cis-regulatory mutations (i.e., co-option of external transcription factors by mutations in existing CREs, co-option of transposable elements as new CREs, loss of transcriptional factors inputs in existing CREs, and remodeling of CREs) that modify timing and threshold responses in caste-specific signalization within a particular GRN but not in others. This results in a change in pattern of differential gene expression among caste-specific modules. In cases where polyphenism is controlled by the social environment, the evolution of the frequencies of intercaste production and the range of mosaics produced could also occur via the selection of genetic determinants of social behaviors, i.e., brood care and food supply [105].

Yang and Abouheif [109] described asymmetric male–female mosaics (gynandromorphs) as monsters with no evolutionary significance, but one possible outcome of symmetric queen-worker mosaics is the evolution of novel castes in ants. For instance, we found that most mosaics have a queen-like head and gaster but worker-like thorax. These phenotypes are congruent with the morphology of ergatoid queens and soldiers, two castes that have repetitively evolved across ants and are suspected to have evolved by phenotypic recombination [69]. Indeed, soldiers recombine a worker thorax (winglessness) with a queen head (defense or seed milling) or a queen gaster (trophic eggs). Similarly, ergatoid queens recombine a worker thorax (winglessness) with a queen gaster (reproductive organs). Therefore, ergatoid queens of *M. oberthueri* [110, 111], a sister species of *M. rogeri*, could have evolved from intercastes produced ancestrally through a process of genetic accommodation [9, 19–22].

## Conclusions

Overall, our results show that the production of reorganized phenotypes can occur as a consequence of modularity and developmental plasticity, due to differential plastic responses among modules. This provides a parsimonious explanation for the propensity of ants to evolve new ergatoid queen and soldier castes because most mosaics phenotypes are congruent with the morphology of these castes. This scenario still needs to be refined by comparing the regulatory gene networks underlying development of both intercastes and novel castes (e.g., [5]), and by studying the behavior of developmental anomalies and quantifying their contribution to colony fitness. More generally, our work underlines the need to take into account developmental plasticity in modern evolutionary thought because it determines which phenotypes can or cannot be produced and thus significantly affects the evolutionary potential of populations.

## Abbreviations

BGA: between group analyses
CREs: cis-regulatory elements
GRNs: gene regulatory networks
PCA: principal component analysis

## References

[1] Moczek AP (2008). On the origins of novelty in development and evolution. BioEssays.

[2] Lewis EB (1963). Genes and developmental pathways. Am Zool.

[3] Gehring WJ, Hiromi Y (1986). Homeotic genes and the homeobox. Annu Rev Genet.

[4] Shubin N, Tabin C, Carroll S (2009). Deep homology and the origins of evolutionary novelty. Nature.

[5] Rajakumar R, San Mauro D, Dijkstra MB, Huang MH, Wheeler DE, Hiou-Tim F, Khila A, Cournoyea M, Abouheif E (2012). Ancestral developmental potential facilitates parallel evolution in ants. Science.

[6] Jacob F (1977). Evolution and tinkering. Science.

[7] Wake DB, Roth G: The linkage between ontogeny and phylogeny in the evolution of complex systems. NewYork: Wiley, 1989.

[8] Duboule D, Wilkins AS (1998). The evolution of ‘bricolage’. Trends Genet.

[9] West-Eberhard MJ: Developmental plasticity and evolution. Oxford University Press; 2003.

[10] West-Eberhard MJ (2005). Developmental plasticity and the origin of species differences. Proc Natl Acad Sci.

[11] West-Eberhard MJ (2005). Phenotypic accommodation: adaptive innovation due to developmental plasticity. J Exp Zoolog B Mol Dev Evol.

[12] Prud’homme B, Minervino C, Hocine M, Cande JD, Aouane A, Dufour HD, Kassner VA, Gompel N (2011). Body plan innovation in treehoppers through the evolution of an extra wing-like appendage. Nature.

[13] Abouheif E, Favé M-J, Ibarrarán-Viniegra AS, Lesoway MP, Rafiqi AM, Rajakumar R. Eco-Evo-Devo: the time has come. In: Landry CR, Aubin-Horth N (eds) Ecological Genomics. Springer Netherlands; 2014:107–125. [Advances in Experimental Medicine and Biology, vol. 781]. 10.1007/978-94-007-7347-9_6 24277297

[14] Davidson EH (1989). Lineage-specific gene expression and the regulative capacities of the sea urchin embryo: a proposed mechanism. Development.

[15] Ray TS (1990). Metamorphosis in the Araceae. Am J Bot.

[16] Raff RA, Kaufman TC: Embryos, genes, and evolution: the developmental—genetic basis of evolutionary change. 1991(XXVI):395.

[17] Foster SA, Baker JA (2004). Evolution in parallel: new insights from a classic system. Trends Ecol Evol.

[18] Waddington CH (1953). Genetic assimilation of an acquired character. Evolution.

[19] Suzuki Y, Nijhout HF (2006). Evolution of a polyphenism by genetic accommodation. Science.

[20] Moczek AP (2007). Developmental capacitance, genetic accommodation, and adaptive evolution. Evol Dev.

[21] Nijhout HF, Suzuki Y (2008). Environment and genetic accommodation. Biol Theory.

[22] Suzuki Y, Nijhout HF (2008). Genetic basis of adaptive evolution of a polyphenism by genetic accommodation. J Evol Biol.

[23] Debat V, David P (2001). Mapping phenotypes: canalization, plasticity and developmental stability. Trends Ecol Evol.

[24] Whitman D, Agrawal A. What is phenotypic plasticity and why is it important. Phenotypic Plastic Insects. 2009; 1–63.

[25] Gilbert SF, Epel D: Ecological developmental biology. Edition 1. Sunderland: Sinauer Associates; 2009.

[26] Nijhout HF (2003). Development and evolution of adaptive polyphenisms. Evol Dev.

[27] Woltereck R (1909). Weitere experimentelle untersuchungen über Artänderung, speziell über das Wesen quantitativer Artunterschiede bei Daphniden. Z Für Indukt Abstamm-Vererbungslehre.

[28] Stearns SC (1989). The evolutionary significance of phenotypic plasticity. Bioscience.

[29] Sultan SE, Bazzaz FA (1009). Phenotypic plasticity in *Polygonum persicaria*. I. Diversity and uniformity in genotypic norms of reaction to light. Evolution.

[30] Thompson DB (1992). Consumption rates and the evolution of diet-induced plasticity in the head morphology of *Melanoplus femurrubrum* (Orthoptera: Acrididae). Oecologia.

[31] Thompson (1999). Genotype–environment interaction and the ontogeny of diet-induced phenotypic plasticity in size and shape of *Melanoplus femurrubrum* (Orthoptera: Acrididae). J Evol Biol.

[32] Oster GF, Wilson EO. Caste and ecology in the social insects. Princeton University Press; 1978. 740003

[33] Snell-Rood EC, Van Dyken JD, Cruickshank T, Wade MJ, Moczek AP (2010). Toward a population genetic framework of developmental evolution: the costs, limits, and consequences of phenotypic plasticity. BioEssays.

[34] Scoville AG, Pfrender ME (2010). Phenotypic plasticity facilitates recurrent rapid adaptation to introduced predators. Proc Natl Acad Sci.

[35] Shaw KA, Scotti ML, Foster SA (2007). Ancestral plasticity and the evolutionary diversification of courtship behaviour in threespine sticklebacks. Anim Behav.

[36] Wund MA, Baker JA, Clancy B, Golub JL, Foster SA (2008). A test of the “Flexible Stem” model of evolution: ancestral plasticity, genetic accommodation, and morphological divergence in the threespine stickleback radiation. Am Nat.

[37] Ghalambor CK, McKAY JK, Carroll SP, Reznick DN (2007). Adaptive versus non-adaptive phenotypic plasticity and the potential for contemporary adaptation in new environments. Funct Ecol.

[38] Moczek AP, Sultan S, Foster S, Ledón-Rettig C, Dworkin I, Nijhout HF, Abouheif E, Pfennig DW: The role of developmental plasticity in evolutionary innovation. Proc R Soc Lond B Biol Sci 2011; rspb20110971. 10.1098/rspb.2011.0971 PMC3145196 21676977

[39] Wagner GP. Adaptation and the modular design of organisms. In: Morán F, Moreno A, Merelo JJ, Chacón P, editors. Advances in Artificial Life.. Springer Berlin Heidelberg; 1995. p. 315–328. [Lecture Notes in Computer Science, vol. 929].

[40] Breuker CJ, Debat V, Klingenberg CP (2006). Functional evo-devo. Trends Ecol Evol.

[41] Klingenberg CP (2008). Morphological integration and developmental modularity. Annu Rev Ecol Evol Syst.

[42] Klingenberg CP, Leamy LJ, Cheverud JM (2004). Integration and modularity of quantitative trait locus effects on geometric shape in the mouse mandible. Genetics.

[43] Wagner GP, Pavlicev M, Cheverud JM (2007). The road to modularity. Nat Rev Genet.

[44] Burgio G, Baylac M, Heyer E, Montagutelli X (2012). Exploration of the genetic organization of morphological modularity on the mouse mandible using a set of interspecific recombinant congenic strains between C57BL/6 and mice of the *Mus spretus* species. G3 Genes Genomes Genetics.

[45] Carroll SB (2008). Evo-devo and an expanding evolutionary synthesis: a genetic theory of morphological evolution. Cell.

[46] Erwin DH, Davidson EH (2009). The evolution of hierarchical gene regulatory networks. Nat Rev Genet.

[47] Shubin N, Tabin C, Carroll S (1997). Fossils, genes and the evolution of animal limbs. Nature.

[48] Morata G, Garcia-Bellido A (1976). Developmental analysis of some mutants of the bithorax system of Drosophila. Wilhelm Rouxs Arch Dev Biol.

[49] Morata G, Lawrence PA (1979). Development of the eye-antenna imaginal disc of Drosophila. Dev Biol.

[50] Galant R, Carroll SB (2002). Evolution of a transcriptional repression domain in an insect Hox protein. Nature.

[51] Wilson EO: The insect societies. 1971. p. 548.

[52] Hölldobler B, Wilson EO (1990). The Ants.

[53] Fisher BL. AntWeb-Ants of the world. 2012. http://www.antweb.org/ (2015). Accessed 05 Nov 2015.

[54] Heinze J, Tsuji K (1995). Ant reproductive strategies. Res Popul Ecol.

[55] Heinze J, Keller L (2000). Alternative reproductive strategies: a queen perspective in ants. Trends Ecol Evol.

[56] Peeters C, Ito F (2001). Colony dispersal and the evolution of queen morphology in social hymenoptera. Annu Rev Entomol.

[57] Peeters C, Molet M: Chapter 9: colonial reproduction and life histories. In: Lori Lach, Catherine L. Parr, Kirsti L, editors. Abbott, Ant ecology. Oxford University Press; 2010.

[58] Peeters C (2012). Convergent evolution of wingless reproductives across all subfamilies of ants, and sporadic loss of winged queens (Hymenoptera: Formicidae). Myrmecol News.

[59] Cronin AL, Molet M, Doums C, Monnin T, Peeters C (2013). Recurrent evolution of dependent colony foundation across eusocial insects. Annu Rev Entomol.

[60] Molet M, Maicher V, Peeters C (2014). Bigger helpers in the Ant *Cataglyphis bombycina*: increased worker polymorphism or novel soldier caste?. PLoS One.

[61] Wheeler DE (1986). Developmental and physiological determinants of caste in social hymenoptera: evolutionary implications. Am Nat.

[62] Wheeler DE (1991). The developmental basis of worker caste polymorphism in ants. Am Nat.

[63] Hartfelder K, Engels W. Current topics in developmental biology. Elsevier; 1998. 10.1016/s0070-2153(08)60364-6 9673848

[64] Evans JD, Wheeler DE (2001). Gene expression and the evolution of insect polyphenisms. BioEssays.

[65] Moczek AP, Snell-Rood EC (2008). The basis of bee-ing different: the role of gene silencing in plasticity. Evol Dev.

[66] Elango N, Hunt BG, Goodisman MAD, Yi SV (2009). DNA methylation is widespread and associated with differential gene expression in castes of the honeybee, Apis mellifera. Proc Natl Acad Sci.

[67] Abouheif E, Wray GA (2002). Evolution of the gene network underlying wing polyphenism in ants. Science.

[68] Schwander T, Lo N, Beekman M, Oldroyd BP, Keller L (2010). Nature versus nurture in social insect caste differentiation. Trends Ecol Evol.

[69] Molet M, Wheeler DE, Peeters C (2012). Evolution of novel mosaic castes in ants: modularity, phenotypic plasticity, and colonial buffering. Am Nat.

[70] Wheeler WM. Worker ants with vestiges of wings. Order Trutees Am Mus Nat Hist 1905.

[71] Wheeler WM, Weber NA. Mosaics and Other Anomalies among Ants. 1937.

[72] Brian MV (1955). Studies of caste differentiation in «*Myrmica Rubra*» L. Insectes Soc.

[73] Plateaux L: Sur le polymorphisme social de la fourrni *Leptothorax nylanderi* (Förster). I. Morphologie et biologie comparee des castes. 1970.

[74] Peeters CP (1991). Ergatoid queens and intercastes in ants: two distinct adult forms which look morphologically intermediate between workers and winged queens. Insectes Soc.

[75] Heinze J (1998). Intercastes, intermorphs, and ergatoid queens: who is who in ant reproduction?. Insectes Soc.

[76] Wang X, Chamberlin HM (2002). Multiple regulatory changes contribute to the evolution of the *Caenorhabditis* lin-48 ovo gene. Genes Dev.

[77] Gompel N, Prud’homme B, Wittkopp PJ, Kassner VA, Carroll SB (2005). Chance caught on the wing: cis-regulatory evolution and the origin of pigment patterns in Drosophila. Nature.

[78] Bejerano G, Lowe CB, Ahituv N, King B, Siepel A, Salama SR, Rubin EM, James Kent W, Haussler D (2006). A distal enhancer and an ultraconserved exon are derived from a novel retroposon. Nature.

[79] Hinman VF, Davidson EH (2007). Evolutionary plasticity of developmental gene regulatory network architecture. Proc Natl Acad Sci.

[80] Zinzen RP, Cande J, Ronshaugen M, Papatsenko D, Levine M (2006). Evolution of the Ventral Midline in Insect Embryos. Dev Cell.

[81] Düssmann O, Peeters C, Hölldobler B (1996). Morphology and reproductive behaviour of intercastes in the ponerine ant *Pachycondyla obscuricornis*. Insectes Soc.

[82] Okada Y, Plateaux L, Peeters C (2013). Morphological variability of intercastes in the ant *Temnothorax nylanderi*: pattern of trait expression and modularity. Insectes Soc.

[83] Molet M, Fisher BL, Ito F, Peeters C (2009). Shift from independent to dependent colony foundation and evolution of “multi-purpose” ergatoid queens in *Mystrium* ants (subfamily Amblyoponinae). Biol J Linn Soc.

[84] Yoshimura M, Fisher BL. A revision of the ant genus *Mystrium* in the Malagasy region with description of six new species and remarks on Amblyopone and Stigmatomma (Hymenoptera, Formicidae, Amblyoponinae). ZooKeys. 2014;1–99. 10.3897/zookeys.394.6446 PMC3978267 24715784

[85] Claude J. Morphometrics with R. Springer; 2008.

[86] Rohlf J: tpsDig2. 2009.

[87] Klingenberg CP, McIntyre GS (1998). Geometric morphometrics of developmental instability: analyzing patterns of fluctuating asymmetry with procrustes methods. Evolution.

[88] Bookstein FL (1997). Landmark methods for forms without landmarks: morphometrics of group differences in outline shape. Med Image Anal.

[89] Andresen PR, Bookstein FL, Couradsen K, Ersboll BK, Marsh JL, Kreiborg S (2000). Surface-bounded growth modeling applied to human mandibles. IEEE Trans Med Imaging.

[90] Sheets HD, Kim K, Mitchell CE, Elewa DAMT (2004). A combined landmark and outline-based approach to ontogenetic shape change in the Ordovician trilobite *Triarthrus becki*. Morphometrics.

[91] Klingenberg CP, Barluenga M, Meyer A (2002). Shape analysis of symmetric structures: quantifying variation among individuals and asymmetry. Evolution.

[92] Goodall C (1991). Procrustes methods in the statistical analysis of shape. J R Stat Soc Ser B Methodol.

[93] Smith DR, Crespi BJ, Bookstein FL (1997). Fluctuating asymmetry in the honey bee, *Apis mellifera*: effects of ploidy and hybridization. J Evol Biol.

[94] Adams DC, Otárola-Castillo E (2013). Geomorph: an r package for the collection and analysis of geometric morphometric shape data. Methods Ecol Evol.

[95] Culhane AC, Perrière G, Considine EC, Cotter TG, Higgins DG (2002). Between-group analysis of microarray data. Bioinformatics.

[96] Doledec S, Chessel D (1987). Rythmes saisonniers et composantes stationnelles en milieu aquatique. I: Description d’un plan d’observation complet par projection de variables. Acta Oecologica Oecologia Gen.

[97] Fukunaga K. Introduction to statistical pattern recognition. Academic Press; 1990.

[98] Efron B, Tibshirani R (1986). Bootstrap methods for standard errors, confidence intervals, and other measures of statistical accuracy. Stat Sci.

[99] Klingenberg CP: Multivariate allometry. In Marcus LF, Corti M, Loy A, Naylor GJP, Slice DE, editors. Advances in Morphometrics. USA: Springer; 1996. p. 23–49. [NATO ASI Series, vol. 284].

[100] Fairbairn DJ (1997). Allometry for sexual size dimorphism: pattern and process in the coevolution of body size in males and females. Annu Rev Ecol Syst.

[101] Schwander T, Humbert J-Y, Brent CS, Cahan SH, Chapuis L, Renai E, Keller L (2008). Maternal effect on female caste determination in a social insect. Curr Biol.

[102] Brian MV (1951). Caste determination in a *Myrmicine* ant. Experientia.

[103] Windig JJ (1994). Reaction norms and the genetic basis of phenotypic plasticity in the wing pattern of the butterfly *Bicyclus anynana*. J Evol Biol.

[104] Murakami T, Ohkawara K, Higashi S (2002). Morphology and developmental plasticity of reproductive females in *Myrmecina nipponica* (Hymenoptera: Formicidae). Ann Entomol Soc Am.

[105] Linksvayer TA, Kaftanoglu O, Akyol E, Blatch S, Amdam GV, Page RE (2011). Larval and nurse worker control of developmental plasticity and the evolution of honey bee queen–worker dimorphism. J Evol Biol.

[106] Scheiner SM (1993). Genetics and evolution of phenotypic plasticity. Annu Rev Ecol Syst.

[107] Nicoglou A (2015). The evolution of phenotypic plasticity: genealogy of a debate in genetics. Stud Hist Philos Sci Part C Stud Hist Philos Biol Biomed Sci.

[108] Moczek AP (2003). The behavioral ecology of threshold evolution in a polyphenic beetle. Behav Ecol.

[109] Yang AS, Abouheif E (2011). Gynandromorphs as indicators of modularity and evolvability in ants. J Exp Zoolog B Mol Dev Evol.

[110] Molet M, Peeters C, Fisher BL (2007). Winged queens replaced by reproductives smaller than workers in *Mystrium* ants. Naturwissenschaften.

[111] Molet M, Peeters C, Follin I, Fisher BL (2007). Reproductive caste performs intranidal tasks instead of workers in the Ant *Mystrium oberthueri*. Ethology.

[112] McGinnis W, Krumlauf R (1992). Homeobox genes and axial patterning. Cell.

[113] Hall BK (2003). Evo-Devo: evolutionary developmental mechanisms. Int J Dev Biol.

[114] Maeshiro T, Kimura M (1998). The role of robustness and changeability on the origin and evolution of genetic codes. Proc Natl Acad Sci.

